# Phenology of *Oithona similis* demonstrates that ecological flexibility may be a winning trait in the warming Arctic

**DOI:** 10.1038/s41598-021-98068-8

**Published:** 2021-09-20

**Authors:** Kaja Balazy, Rafał Boehnke, Emilia Trudnowska, Janne E. Søreide, Katarzyna Błachowiak-Samołyk

**Affiliations:** 1 Department of Marine Ecology, Institute of Oceanology, Polish Academy of Sciences, Powst. Warszawy 55, 81-712 Sopot, Poland; 2 Department of Arctic Biology, The University Centre in Svalbard (UNIS), PB 156, 9171 Longyearbyen, Norway

**Keywords:** Ecology, Ecology

## Abstract

Rapidly warming Arctic is facing significant shifts in the zooplankton size-spectra manifested as increasing numbers of the small-sized copepod *Oithona similis*. Here we present a unique continuous data set covering 22 months, on its copepodite structure along with environmental drivers in the Atlantic-influenced high Arctic fjord Isfjorden (Spitsbergen). Abundance maxima of *O*. *similis* were observed in September when the highest seawater temperature was recorded. A high concentration of the indicator species of Atlantification *Oithona atlantica* was also observed at that time. The clear dominance of *O*. *similis* in the zooplankton community during the dark, theoretically unproductive season emphasizes its substantial role in sustaining a continuous carbon flow, when most of the large herbivorous copepods fall into sleeping state. The high sex ratio observed twice in both years during periods of high primary production suggests two main reproductive events per year. *O*. *similis* reproduced even in very low temperatures (< 0 °C) previously thought to limit their fecundity, which proves its unique thermal tolerance. Our study provides a new insight on ecology of this key copepod of marine ecosystems across the globe, and thus confirm the Climatic Variability Hypothesis assuming that natural selection favour species with such flexible adaptive traits as *O*. *similis*.

## Introduction

Climate change is likely to have major impacts on global ecosystems, and this is especially the case in the rapidly warming Arctic regions^1,2^. Warmer sea temperatures and a longer open water season create new opportunities for sub-Arctic and boreal species to establish themselves in the high Arctic and these may outcompete those Arctic species currently living there^3–6^. Systematic reorganization of zooplankton communities from larger Arctic to tiny boreal/temperate taxa has already been observed along the west coast of Spitsbergen, with a particular increase in numbers of the small cosmopolitan copepods *Oithona* spp.^7–9^. The scale of this phenomenon is mainly influenced by the high numbers of *O*. *similis*, while much less numerous *O*. *atlantica* is typically regarded as the key representative of Atlantic expatriates and is often used as an indicator of Atlantic inflow^10^. Despite its low individual biomass, the high abundance of *O*. *similis* can contribute considerably to both biomass and secondary production, especially in coastal and fjord waters of the northern hemisphere^11–13^. Recently it was demonstrated that in fjords prone to intensive Atlantic water advection, the increase of abundance of *O*. *similis* results in a change in the zooplankton size spectra^14^. The 7-year studies from the Arctic shelf region with measurements repeated at the same time of the year (July/August), clearly confirmed that higher water temperatures favour the small zooplankton fractions represented (among others) by *O*. *similis* over larger ones^15^.

What makes *O*. *similis* particularly flexible and able to adapt to changing environmental conditions is its thermal plasticity and omnivorous feeding strategy^16^. The temperature tolerance from 4 °C to even > 20 °C^17,18^ is wider than in any other copepods species potentially explaining its ubiquity in the world oceans^16^. *O*. *similis* exploit the lower portion of the food size spectrum than large herbivorous copepods and feeds primarily upon ciliates and heterotrophic dinoflagellates as well as on heterotrophic protists or even copepod nauplius^16,19,20^. Since its diet is coupled more with microbial loop than to phytoplankton blooms it is not strictly constrained by the seasonal restriction in primary production as large herbivorous copepods^16^. All these abilities allow *O*. *similis* to maintain an almost-continuously stable population and stay active even over polar night when primary production is severely reduced. Although new studies show that *Calanus* spp. can also be active in winter^21–23^, this period clearly belongs to small copepods, which due to their opportunistic feeding act as a main microbial grazers, sustaining continuous flow and utilizing of either primary or regenerated production^24^.

Although *O*. *similis* has been widely studied, there are inherent limitations that make it difficult to accurately trace its population dynamics. One of the main problems is time limitation. Previous studies on *O*. *similis* from Svalbard have largely been limited to the summer seasons. There have only been a few seasonal studies on zooplankton including *O*. *similis* carried out in Spitsbergen fjords using nets: in 80 s^25^, and 90 s, when the fjords on the west coast of Svalbard still experienced cold Arctic conditions and seasonal sea ice cover^12,26,27^; and more recently from data from sediment traps^28^. These studies did not specifically consider the entire population structure of *O. similis*. Similar limitations apply to studies from other polar regions such as Western Greenland^29,30^, the Barents Sea^31^ or Northern Norway^32^. Although most of these studies looked at the *O*. *similis* complete age structure, they did not cover all of the seasons, and this has limited our interpretation of the full annual cycle. Another important limitation is the sampling method, as only the adults are representatively sampled in the standard, zooplankton surveys nets of 180–200 µm mesh sizes^33–35^ commonly used in high latitudes, which is why the information on its complete population structure in the Arctic is relatively poor. Böttger-Schnack et al.^36^ found that the abundances of *Oithona* spp. recorded for younger copepodite stages and adults differed by a factor of 3 to 7 when comparing zooplankton net samples with mesh sizes 150 versus 55 μm. It is thus likely that the reported abundances of *O. similis* may be severely underestimated in most zooplankton studies^24^. In temperate climatic conditions the abundance and reproductive output of *O*. *similis* is strongly related to the variation in sea surface temperature (SST)^37^. However, the high seasonality of heterotrophic protists associated with primary production peaks observed at high latitudes may suggest that the food availability is also an important factor influencing population dynamics of *O*. *similis* in these regions^38–40^. Even though previous studies performed in Greenland implied that *O*. *similis* is active year-round and has the ability to reproduce even in the food-limited conditions^29,30^, we assumed that their functioning in other, Atlantic-influenced regions may exhibit a different pattern. It was interesting to test it in region considered as an indicator for climate change in the European Arctic^41^ over two different years to verify which of the patterns/processes are stable regardless of the environmental conditions, and which are more prone to changes in environmental settings. In this study, we present a unique phenological study of *O*. *similis*, based on high temporal resolution sampling that was performed weekly/monthly during two successive years, from January 2012 to October 2013 by a fine-meshed (63 µm) zooplankton net in Adventfjorden, Isfjorden (Svalbard). Since 2005 this fjord has experienced strong Atlantic water influence and remained ice free. The aim of this study was to follow the temporal dynamics of *O*. *similis* copepodite structure in the mesozooplankton community over the course of 22 months and to test how it relates to dynamically changing physical and biological environmental conditions. We hypothesized that because of the boreal nature of *O*. *similis*^16^ and that cold temperature act as a limiting factor of its fecundity^42^, the increase in the population of *O*. *similis* will be associated with higher seawater temperature. We also expect that due to being broadly omnivorous^16^, and because of the known ability of *O*. *similis* to reproduce year-round^43^, *O*. *similis* would be capable of continuous reproduction also in such an high Arctic region, however, due to the exceptional seasonality of the environment, a pattern of increased reproductive activity and abundance, driven by biotic and abiotic factors will be distinguished, differently to more stable populations from temperate climates^32^.

## Material and methods

### The study area

Adventfjorden is a small (around 7 km long and 4 km wide) NW–SE directed side arm of Isfjorden, the largest fjord complex in west Spitsbergen. Isfjorden and Adventfjorden are open fjords with no physical sill barriers and are seasonally influenced by warm Atlantic water and transformed Atlantic water transported/advected by the West Spitsbergen Current from the shelf outside, as well as by local river run-offs^41,44^. Adventfjorden is located at a latitude of 78° N where the sun remains below the horizon for almost 4 months during the winter and stays above the horizon for 4 months during summer, providing a characteristic high Arctic light climate^45^. However, because of the hydrography and the lack of seasonal ice cover during winter in recent years, Adventfjorden is regarded as being more typical of a sub-Arctic fjord^46^.

**Figure 1:**
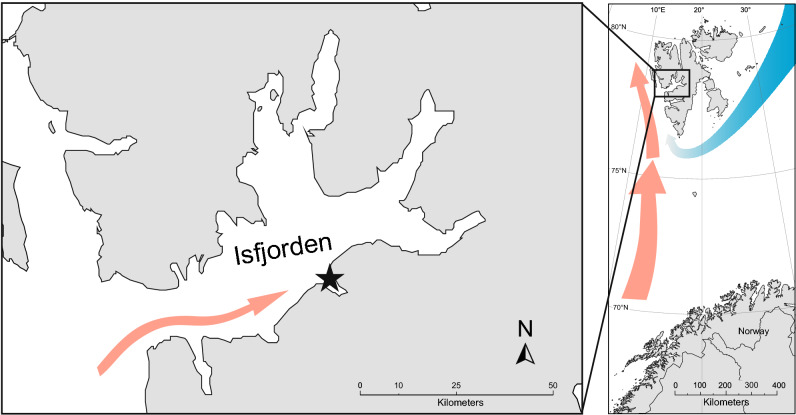
Location of the sampling station Isfjorden/Adventfjorden IsA (black star), Spitsbergen, Norway. The arrows show the dominating ocean currents: warm West Spitsbergen Current (red arrow) advected to the southern part of the Isfjorden and cold Sørkapp Current (blue arrow). Map was prepared with Ocean Data View software v. 5.0.0 (http://odv.awi.de)^61^.

The time-series zooplankton sampling was performed at Isfjorden-Adventfjorden station (IsA, 78.261 N, 15.535 E, Fig. 1), which is located near the mouth of Adventfjorden and is 80 m deep. The close location of the University Centre in Svalbard (UNIS) has enabled a full-year measurement campaign, conducted in 2012 (January–December) and 2013 (January–October).

### Environmental parameters

Environmental parameters (temperature and salinity) were measured using a hand-held CTD (SAIV SD204, Bergen, Norway) with an attached Seapoint fluorometer or by using a Seabird 911 ship CTD. In this study the average for seawater temperature (hereafter ST) and salinity was calculated for a 0–65 m water layer. Chlorophyll *a* concentration was estimated from water samples collected at 5, 15, 25 and 60 m with a 10 L Niskin bottle. Three replicates were filtered through glass microfiber filters (GF/F, 0.7 mm, Whatman, England). Filters were either stored frozen (-80 °C) or chlorophyll *a* was extracted immediately in 10 mL methanol (~ 99%) for 20–24 h at 4 °C in darkness^47^. Chlorophyll *a* concentrations were measured with a calibrated fluorometer (10-AU-005-CE Fluorometer, Turner, USA; chl *a* standard:Sigma S6144). The replicate samples were used to calculate the average chlorophyll concentration for 0–65 m water layer. More detailed data on the specific environmental conditions from 2012 can be found in Stübner et al.^48^. Average day-length was calculated using the NOAA solar calculator available at http://www.esrl.noaa.gov/gmd/grad/solcalc/calcdetails.html, using the hours per day where the sun was above the horizon for each day of sampling. For more details on chlorophyll *a* values and protist diversity see Marquardt et al.^49^ and Kubiszyn et al.^38^.

### Zooplankton sampling and taxonomic analysis

Zooplankton samples were collected from the IsA sampling station one to four times per month between January 2012 and October 2013^48^. Zooplankton data from the year 2012 was partly presented in Stübner et al.^48^. Sampling was more frequent from the beginning of March until the end of May, when the spring bloom was observed in both of the studied years. Two vertical hauls of a closing WP2 net with 63 µm mesh size and opening area of 0.25 m^2^^50^ were conducted from 25–0 and 65–25 m at a rate of 0.25–0.5 ms^-1^. Samples were preserved with 4% borax buffered formaldehyde-seawater solution. In the laboratory, detailed taxonomic analysis was performed according to standard procedure. First, in each sample macrozooplankton (with total body length ≥ 5 mm) was removed, identified and counted. Then, for remaining mesozooplankton, 2 ml sub-samples were taken and all the organisms were identified to the lowest possible taxonomic level following Harris et al.^51^. Sub-samples were identified until a total number of 400 of all individuals from different taxa were counted. After sample analysis, sub samples with a special focus on the copepodite composition of *O*. *similis* (adult female—AF, adult male—AM, fifth copepodite stage—CV, fourth copepodite stage—CIV, third copepodite stage—CIII, second copepodite stage—CII, first copepodite stage—CI) were examined. From each sample, 2 ml subsamples were taken to count at least 50 individuals of *O*. *similis*. Where there was a low abundance of *O*. *similis* samples were divided into two parts using a Motodo plankton splitter and every individual of *O. similis* from one half was identified to an appropriate developmental stage.

### Data analysis

We employed the phenological indices commonly applied to stage-structured abundance data as recently applied to *Calanus*^52^. This involved calculating the proportion of copepodite stages V (CV) to the total abundance of copepodite stages (CVT), as described by Mackas et al.^53^. The population development index (PDI) was calculated as the proportion of early copepodite stages (CI to CIII) to total abundance of copepodites according to Head et al.^54^. The average weighted stage (AWS) was calculated on the basis of relative abundance of particular life stages, with each stage multiplied by values from 1 for CI to 6 for adults^55^. The sex ratio of adult males/adult females (AM/AF) was expressed as a relative number of males to females^56^. The value of AM/AF ≥ 0.12 was used as a determinant of reproduction events as characteristic for the female-skewed Oithonidae family^56^. Since all these phenological indices represent different stages and various aspects of life cycle, we applied them together to provide a more complete overview of the *O*. *similis* phenological variability^52,57^. This approach is especially relevant for seasonal study with high dynamics of population variability as in our research^52^. Multivariate nonparametric permutational ANOVA (PERMANOVA)^58^ was used to test differences in the zooplankton taxonomic composition based on abundances of each species/life stage identified, the monthly mean abundance of *O*. *similis*, the concentration of individual copepodite stages in particular water layer and the copepodite structure based on the abundance of each stage between particular months. Prior to the analyses, abundance data were square-root-transformed^59^. The calculation of the Pseudo-F and p values was based on 999 permutations of the residuals under a reduced model^60^. The distribution of centroids representing particular samples was illustrated with a non-metric multi-dimensional scaling (nMDS) using Bray–Curtis similarity ordination to demonstrate variability in zooplankton community structure. The relationship between the proportion of *O*. *similis* total abundance and proportion of *Calanus* spp. in total copepod abundance (TCA) was tested using Pearson linear correlation. A distance-based linear model (DistLM) was used to analyse the relationships between the total abundance, CVT, AWS, AM/AF and PDI vs. the environmental variables including temperature, salinity, day length, and chlorophyll *a* concentration. *Calanus* spp. abundance was also treated as an environmental factor. Prior to the analyses, *Oithona* spp. abundance and phenological parameters (CVT, PDI, AWS, AM/AF) were square-root-transformed and the environmental variables were normalized. A forward-selection procedure was used to determine the best combination of predictor variables explaining variations in abundance and phenological indices. The selection criteria were based on R^2^ values^58^. The Ocean Data View software programme^61^ was used to prepare maps.

## Results

### Environmental variability

**Figure 2:**
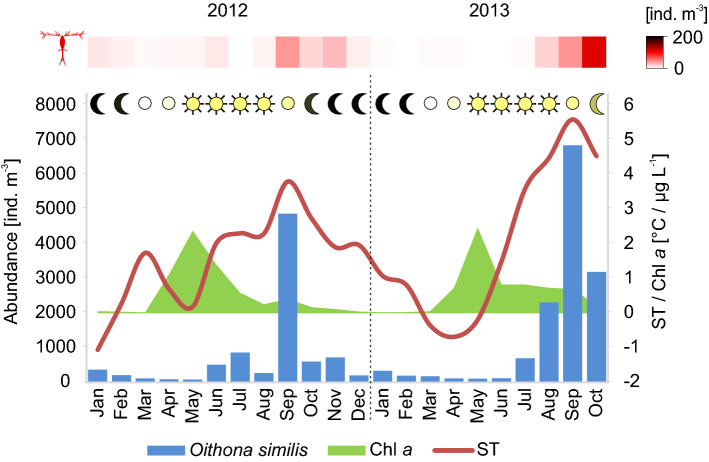
*Oithona similis* monthly mean abundance (ind. m^-3^) (left Y axis); monthly mean chlorophyll *a* concentration (Chl *a*, µg L^-1^) and seawater temperature (ST, °C) (right Y axis). The *O*. *atlantica* monthly mean abundance (ind. m^-3^) is represented by different shades of red (upper panel) measured at IsA station from 0–65 m and the monthly mean day-length (moon/sun icon) is expressed by different shades of yellow calculated using the NOAA solar calculator.

The water masses in Adventfjorden, were found to be relatively well mixed for most of the year in 2012 and 2013 except during the period between July–September when local freshwater run-off formed a distinct fresher and warmer surface water layer. The local waters dominated with pulses of modified Atlantic water in March–April in 2012 and in June in 2013. Seawater temperatures (ST) remained above zero most of the year except for January–February 2012 and March–May in 2013 (Fig. 2). In both years the highest ST was recorded in September with 3.8 °C in 2012 and 5.6 °C in 2013 (Fig. 2). The lowest ST was observed in January (− 1.1 °C) in 2012 with a clear drop observed also in May (Fig. 2) and in April (-0.7 °C) in 2013. Salinity was highest during February-April (34.7–34.8) while the lowest values were observed during July–September (33.6—34.1). The sun was above the horizon for 24 h from the end of April until August. Day lengths become rapidly shorter from September until complete darkness lasting from November to February (Fig. 2). The spring bloom, expressed by elevated chlorophyll *a* values were recorded in May for both years (2.4 and 2.5 mg Chla L-1 in 2012 and 2013, respectively, Fig. 2), and lasted until July. The second, though much less pronounced peak of chlorophyll *a* occurred in the autumn from August to October (0.3–0.4 mg Chla L-1 in 2012 and 0.7–0.8 mg Chla L-1 in 2013). Thereafter the chlorophyll *a* values were close to zero until March (Fig. 2).

### Abundance and proportional importance of Oithona

The mean abundances of *O*. *similis* differed between the months studied (PERMANOVA, *MS* = 1254.8, *Pseudo-F* = 4.61, *P* = 0.011) but did not differ between years (PERMANOVA, *MS* = 241.7, *Pseudo-F* = 0.89, *P* = 0.376). The most pronounced peaks of *O*. *similis* (monthly mean abundances) were observed in September in both years (Fig. 2). The environmental variables tested (salinity, temperature, chlorophyll *a* concentration, day length, *Calanus* spp. abundance) explained 74% of the total variability in the abundance of *O*. *similis*. The variation in seawater temperature (ST) best explained the observed abundance variability (62%) (Supplementary Table S1), and indeed peaks of *O*. *similis* clearly coincided with the highest temperature recorded in both years (Fig. 2). The highest abundances of *O*. *atlantica* were noted in September 2012 and September–October 2013 (Fig. 2).

**Figure 3:**
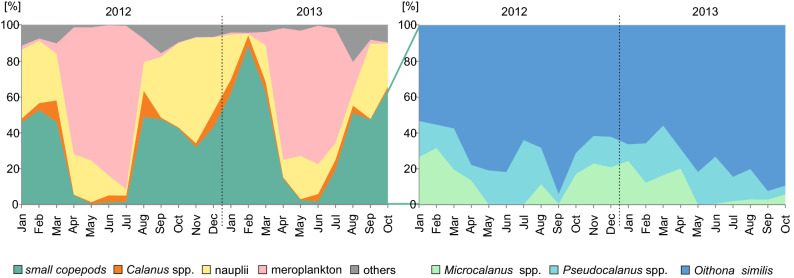
Percentage of the abundance of small copepods in comparison with other dominant groups (left graph) and share (%) of *Microcalanus* spp., *Pseudocalanus* spp. and *Oithona similis* to the small copepods (right graph) over 22 studied months in Adventfjorden.

The zooplankton community composition showed clear seasonal pulses with the highest numerical importance of small copepods found during the autumn and winter months, whereas other mesozooplankton taxa, represented mainly by meroplankton (larval stages of Bivalvia and Cirripedia) predominated during the spring–summer period (Fig. 3). *O*. *similis* was the dominant species among the small copepods in all seasons in both years (Fig. 3). The two other small copepods taxa *Pseudocalanus* spp. and *Microcalanus* spp. were the next most abundant and were usually observed in similar numbers, except for slightly higher values for *Microcalanus* spp. during the winter months, and a stronger dominance of *Pseudocalanus* during spring/summer months especially during May–July 2012 and May 2013 when *Microcalanus* spp. was not recorded (Fig. 3).

**Figure 4:**
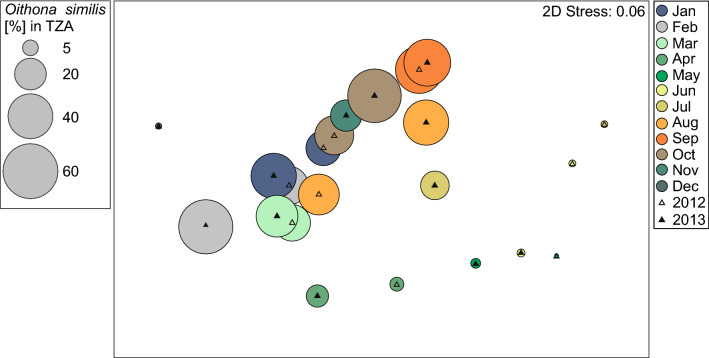
Two-dimensional non-metric multidimensional scaling (nMDS) ordination of the overall similarity in zooplankton taxonomic composition (the closer the points the more similar species composition). The relative sizes of the bubbles represents the share (%) of *Oithona similis* related to the total zooplankton abundance (TZA).

**Figure 5:**
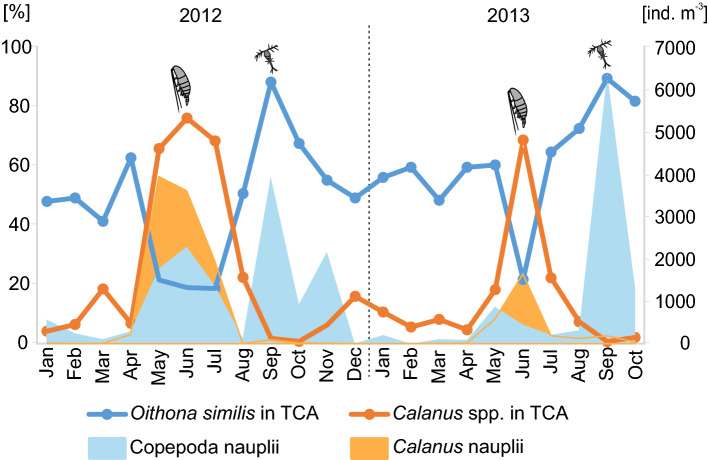
*Oithona similis* and *Calanus* spp. relative abundance (%) in Total Copepoda Abundance (TCA) and total abundance (ind. m^-3^) of Copepoda nauplii and *Calanus* nauplii.

The zooplankton taxonomic composition was similar in the corresponding months of the two studied years (PERMANOVA, *MS* = 1119.7, *Pseudo-F* = 1.622, *P* = 0.163) (Fig. 5). Significant differences were found between individual months (PERMANOVA *MS* = 2814.8, *Pseudo-F* = 4.0774, *P* = 0.001). Numerically, *O. similis* constituted an important component (25% on average) of the overall zooplankton community, especially during winter and autumn months with shares of up to 60% noted in February 2013 and October 2013 (Fig. 4). This high share (~ 45%) was also noted in both years in September. In contrast, in spring and summer months the percentage of *O*. *similis* was found to be relatively low (< 5%).

The proportion of *O*. *similis* to total Copepoda abundance (TCA) including both small and large copepods was negatively correlated with proportion of *Calanus* spp. in TCA (Pearson correlation, *r* = -0.84, *P* < 0.001). *Calanus* spp. was the dominant taxon among copepods during the summer months (almost 80% and 70% in June 2012 and July 2013, respectively), while the highest proportion of *O*. *similis* was noted during the autumn/winter months (almost 90% in September 2012 and November 2013, respectively, Fig. 5). Copepod nauplii including naupliar stages of small copepods such as *Pseudocalanus* spp., *Acartia* spp. and *Oithona* spp. peaked 2–3 times a year with the highest abundances observed in September in both years (Fig. 5). In turn *Calanus* nauplii peaked only once a year in May/June (Fig. 5).

### Oithona population development and the effect of environmental factors

In general, the population of *O*. *similis* was concentrated in the surface water layer (0–25 m), which was particularly pronounced in September in both years. Slightly higher abundances of *O*. *similis* in the bottom layer (65–25) were only observed in July 2012 and March, June and October of 2013 (Supplementary Fig. S1). Generally, there were no differences and no clear preferences of particular copepodite stages to specific water layers (PERMANOVA, *MS* = 1102.8, *Pseudo-F* = 1.04, *P* = 0.327).

**Figure 6:**
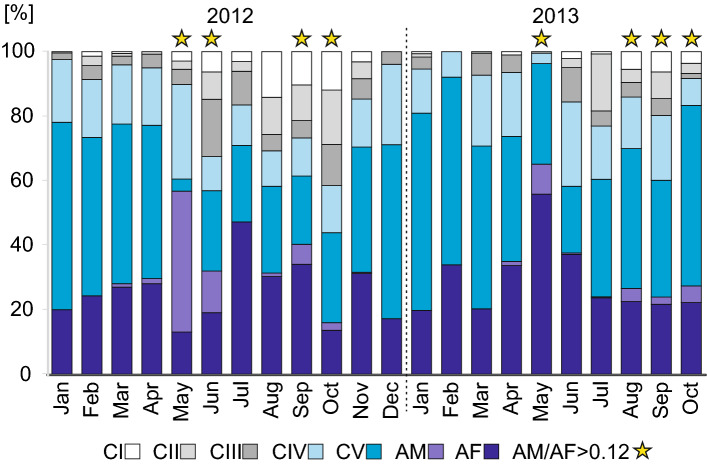
*Oithona similis* copepodite stages (CI–CV, AF–adult females, AM–adult males) copepodite stage structure in integrated water column (0–65 m). Yellow stars indicate sex ratio (AM/AF) higher than 0.12^56^.

The copepodite structure of *O*. *similis* with grouped stages (early CI-CIII, late CIV-CV, adult females AF, and adult males AM) was similar in corresponding months of the two studied years (PERMANOVA, *MS* = 199.9, *Pseudo-F* = 2.20, *P* = 0.129) but the factor of month was statistically significant (PERMANOVA, *MS* = 218.0, *Pseudo-F* = 2.40, *P* = 0.023). The highest proportion of early copepodite stages (CI-CIII) was observed from June to October in both years with the maximum proportion of about 40% observed in October 2012 (Fig. 6). Copepodites CIV-CV of *O*. *similis* were the dominant stages for most of the year with the highest proportions during the winter/early spring months (Fig. 6). The highest proportion of AF was observed in May 2012 with about 47% and in July 2013 with almost 56%. Distinct peaks in the proportion of AM in *O*. *similis* copepodite composition were observed twice a year, in spring and summer, and in autumn and winter in both years (Fig. 6). Sex ratios higher than 0.12^56^ were observed twice a year, in May/June and September/October in both years.

**Figure 7:**
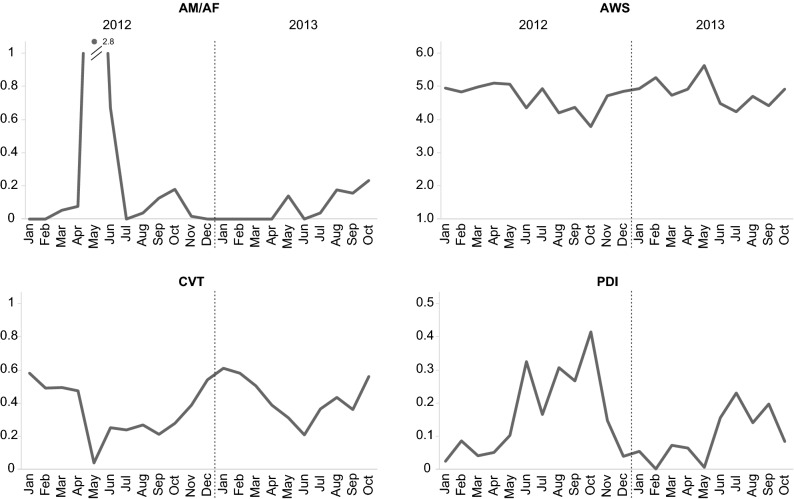
*Oithona similis* phenological indices: sex ratio (AM/AF), average weighted stage (AWS), the proportion of copepodite stages V to total abundance of copepodite stages (CVT) and population development index (PDI).

All tested environmental variables explained 62% of the total variability in sex ratio (AM/AF) of *O. similis*, with the chlorophyll *a* concentration having the highest impact (42%) (Supplementary Table S1). In general, AM/AF peaks were associated with higher chlorophyll *a* values, matching in time with spring and autumn phytoplankton blooms. In 2012 AM/AF was especially high (2.8), with an extreme peak observed in May/June (Fig. 7). The DistLM procedure confirmed a significant effect of environmental variables on average weighted stage (AWS) total variability. The greatest effect on the observed variations (42%) in AWS had ST (Supplementary Table S1), with mostly higher AWS values associated with lower ST. In general terms, AWS remained relatively high (> 4) during most of the time in both years. In 2012 the lowest AWS was recorded in October, while the highest values (> 5) were observed during spring (Fig. 7). In turn, in 2013, high AWS values were recorded in February and May and the lowest values were recorded in July (Fig. 7). Environmental variables explained 50% of the total variability in CVT, with the chlorophyll *a* concentration as the factor having the highest statistically significant impact (43%) (Table S1). Generally, the highest CVT was associated with the lowest chlorophyll *a* concentration. In 2012 CVT was the lowest during May and September with the highest values noted during the winter months. Similarly, in 2013 the CVT was lower in June and September with peaks observed during winter months (Fig. 7). The DistLM procedure also confirmed a significant effect of environmental variables on the PDI total variability (48%) with the greatest effect of the observed variation explained by ST (31%). PDI typically raised in line with an increase in ST, especially during the summer and autumn months (Fig. 7).

## Discussion

The increasing advection of Atlantic waters^62^ has been reported to bring warm-water species into the Atlantic sector of the Arctic^63,64^, and to cause a general selection towards smaller species in communities^65^, which has already been demonstrated in copepods^66,67^. Contrary to worries of reduced nutritional value, induced by switch from arctic to boreal zooplankton species, the results of a study by Renault et al.^3^ argue, that these transformations may provoke even a more efficient way of energy transfer in arctic food webs. However, this scenario only assumes shift within the *Calanus* species, while the authors point out that replacement of *Calanus* spp. with much smaller copepods such as *O*. *similis*, may prove to be harmful in terms of available carbon and lipid content for higher trophic levels^68^. However, we must bear in mind that due to high variability in population responses from different regions affected by local physical environment^66^, the true consequences of the zooplankton community reorganization are difficult to generalized^6^. That is why it is so important to conduct research in places most exposed to climate change, especially on the species phenology, which is one of the first affected by temperature change^69^. By thoroughly examining the trends and factors shaping the population dynamics of *O*. *similis*, which presence in the Arctic is becoming increasingly pronounced, our research can contribute to better predicting ecological responses to climate change that will shape the Arctic ecosystems in the future. The Climatic Variability Hypothesis assumes that natural selection would favour species with flexible adaptive traits allowing them to withstand the challenging conditions of fluctuating environments^70^. Good illustration of such hypothesis is this seasonal Arctic research which clearly demonstrated fluctuation of *O*. *similis* population continuously throughout almost 2 years. Although low temperature (< 5 °C) has been previously recognized as an important limiting factor for *O*. *similis* fecundity and distribution^42^, the high abundance of this copepod and indicators being a proxy of its reproductive activity (eg. sex ratio) at much lower temperatures (< 0 °C) during our research suggests, that the adaptation possibilities of this species are much wider than assumed. This indicates that *O*. *similis* is able to be active and maintain a continuous stable population even in the contrasting conditions of a highly seasonal Arctic environment. Additionally, it seems to benefit from Atlantification by increasing its abundance under the highest seawater temperature (Fig. 2). Our research confirms that *O*. *similis* can increase its reproductive output and thereby strengthen its position in the zooplankton community with increasing temperature as part of the large-scale biogeographical changes in the arctic pelagic ecosystems^67,71,72^.

In our study the *O*. *similis* population showed clear seasonal pulses in abundance with particularly high numbers during autumn–winter months and with distinctive peak numbers recorded in September. The observed autumn peak is in line with other seasonal studies on *O. similis* from Svalbard, regardless of the more Arctic or Atlantic character of the studied region^12,27,73^. However, its overall population concentrations are much lower in colder fjord^14,74,75^ than in northern, warmer locations^12^. In contrast, seasonal peaks of *O*. *similis* in lower latitudes are quite different. In the western English Channel, increased numbers of these copepods are also observed in the autumn months, but a clear peak in both adult and copepodite stages occurs annually in spring, especially in March^37,76,77^. Similarly, high abundances of *O*. *similis* in March were also observed in high-latitude Norwegian fjords^32^. In our study, low abundances of *O*. *similis* were observed in spring when the population comprised mainly older copepodite stages, possibly as a result of higher winter mortality^27^. Even though *Oithona* spp. have low mortality rates compared with calanoid copepods^78,79^, they may differ depending on water temperature^80^ and consequently also over seasonal cycles. Some of the observed drastic changes in proportions between life stages (e.g. switch from almost half of the population represented by males in May, and then their negligible concentrations in June) may suggest that the high mortality of some life stages occurred. The main recruitment occurred later on, in June, but was not associated with a drastic increase in the abundance of *Oithona*. The increased mortality of *Oithona* during summer could be related to the decrease in the abundance of ciliates, the preferred prey for *Oithona*^29^, as was also the case in the Barents Sea^80^. It might be also a consequence of a higher predation pressure on small copepods observed generally in summer^81^. Moreover, the summer generation, as developing under a higher temperatures typically has a shorter life cycle, and as under higher competitive regime, may experience higher rates of mortality^31,80^.

High numbers of *O*. *similis* observed in September may be related to the presences of its preferred food (ciliates/heterotrophic protists)^82,83^ after periods of high primary production^38^. It may also take advantage of the microbial loop related to the decomposing of the bloom^16^. In addition, the highest numbers of *O*. *similis* in September corresponded to the highest seawater temperature recorded in this period in both studied years. This may lead to higher hatching success and more successful reproduction and thus recruitment^32,84^. Additionally, increase in abundance of *O*. *similis* coincided with high numbers of *O*. *atlantica* which may indicate stronger Atlantic water influence during that time^10,85^. Interestingly, higher abundances were observed in September 2013 compared to September 2012. This may also be related to a stronger inflow of modified Atlantic water in autumn 2013, or may be explained by local reproduction which is in line with a progressive increase in the number of *O. similis* in a warming Arctic^86^. Considerably higher numbers of *O. similis* are also systematically observed in the warmer fjords of Spitsbergen than those under the influence of cold Arctic currents^11,87^. Interestingly, in Kongsfjorden the population dynamics of *O*. *similis* do not seem to be as related to temperature/advection as in our research^12^. High seasonal dynamics of total abundance observed during this study is also in contradiction with a more stable population abundance observed in lower latitudes^32^.

In our study a clear dominance of *Calanus* spp. over *O*. *similis* in terms of total abundance was observed only during the summer in both of the studied years. This might be a consequence of their different feeding strategies, since these taxa in the Svalbard fjords^9,88,89^ occupy different trophic niches^20,27^. *O*. *similis* clearly prefers the surface layers during most of the year, while the primarily herbivorous *Calanus* species invest in growth and development in spring and summer, add to their lipid reserves in late summer, thereafter ceasing to feed and descending to depth for winter hibernation^90^. Another interesting observation is the increase in the number of *O*. *similis* just after the period of *Calanus* spp. domination, suggesting that this omnivorous species may utilize the regenerated production^91^, or even switch to coprophagous feeding on faecal pellets of the *Calanus*^92^. The more significant numbers of *O*. *similis* observed during autumn and winter (~ 50%) compared to *Calanus* (~ 10%) emphasizes the important role of these small copepods in shaping the pelagic dissolved organic matter (DOM) pool during this time of the year^79^ mainly by fuelling the microbial loop and bacterial growth through sloppy feeding^91^. Our study demonstrated that both taxa dominate the community in an interchangeable way, confirming their different diet preferences and respective roles in the pelagic food-web and/or niche partitioning (adaptation to sharing the environment).

Recent studies have indicated that during the periods of complete darkness in the arctic winter when primary production is close to zero, there is an evidence of unexpectedly high biological activity and unanticipated trophic interactions^93,94^. Evidence from our study suggests that this is an important biological period for the *O*. *similis* population. While its abundances were much lower than in autumn, the relative population share in the mesozooplankton community was very high (~ 60%) during winter, making it the numerically dominant zooplankton species in this period. Small copepods and their naupliar stages numerically dominate the mesozooplankton community during the polar night in other high Arctic Svalbard fjords^12,22,93^, this was also the case in our study, with clear predominance of *O*. *similis*. All this seems to support the earlier studies pointing towards an increasing role of *O*. *similis* in the changing Arctic during the autumn–winter months^24^. Such a high contribution of small copepods during the dark season is especially important for pelagic carbon cycling processes. Although these small copepods can accumulate and utilize lipid reserves during winter^26,95^, they have a different lipid composition when compared to *Calanus* spp.^96^. This suggests that *O*. *similis* is a rather more opportunistic feeder, which may be able to actively use other food sources during low food supply, such as a carnivorous diet and/or faecal pellets of euphausiids^83^. Protozooplankton present at this time of the year^97^ may be an important part of its diet, as indicated by a study on the western coast of Greenland during the winter-spring transition^29^. However, despite these indications, the diet of *O*. *similis* during the polar night in Spitsbergen waters is still not well known.

The ability of *O*. *similis* to reproduce continuously throughout the year reinforces the hypothesis that this is a highly flexible species^98,99^. This may also be the case in our research, as the average weighted stage (AWS) was consistently high (mostly 4–5) and the mean year sex ratio in 2012 and 2013 oscillated near the value of 0.12 as characteristic for a female-skewed Oithonidae family^56^. Regardless of the continuous reproduction, two main spawning periods (indicated by the high sex ratio) occurring during periods with the highest chlorophyll *a* concentrations suggests that *O*. *similis* can have two main generations (G1 and G2) per year. These reproduction events may be linked to phytoplankton dynamics, as also observed in the previous study^12^. Adult females (AF) in our study prevailed in the turn of spring and summer in both years which may suggest that the most productive spawning of *O*. *similis* occurs during this warm period^12^. The second probable spawning period could occur in autumn in both years when high percentage of adult males (AM) and a high sex ratio were observed. This may be an effect of the completed development of the part of a new generation from the first spawning according to the Bêlehràdek temperature function^100^ adapted to *O*. *similis* by Eiane and Ohmann^78^. In turn, the possible spawning in May 2013 could have been the result of the generation reaching maturity from September/October 2012 (calculating according to Eiane and Ohmann)^78^. However, we also need to take into account that some of the second generations of young copepodits may have been advected into Isfjorden with warmer Atlantic water masses since the events of advection may significantly change the age structure of zooplankton community by transporting younger populations^52,101,102^. Nevertheless, *O*. *similis* maintained a continuous population, which indicates that the well-established position of this boreal species in the high Arctic ecosystem may be considered as a sign of further Atlantification of this region^6^.

## Conclusions

**Figure 8:**
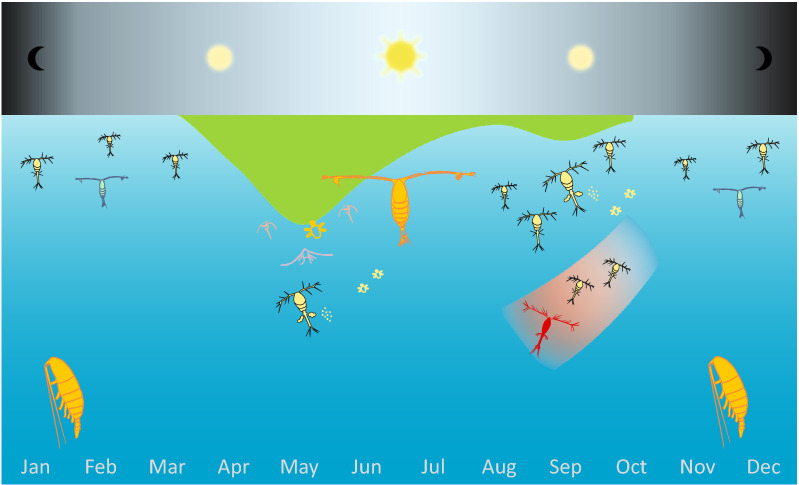
Conceptual figure showing phenology of *Oithona similis* in heavily Atlantic-influenced high Arctic fjord (Isfjorden). *Oithona similis* dominate the mesozooplankton community during autumn/winter together with other small copepods (e.g. *Pseudocalanus* spp.) when larger *Calanus* species descended in depths for winter diapause. In high-spring and summer it avoids overcrowding by meroplankton and herbivores in the upper water layer by peaking in numbers in September after periods of high primary production utilizing post bloom regenerated production. It is possible that the two main reproduction events per year coincide with the periods of high primary production. The abundance peak in September might result both from local reproduction and also be related to a stronger inflow of Atlantic water carrying the Atlantic indicator species—*Oithona atlantica* (marked in red) during that time.

Among the mechanisms that govern *Oithona* population dynamics is not only its omnivorous diet, low mortality, thermal plasticity, but also the frequent reproduction and the ability to take over the scene at the right moment. Our study indicated that the timing of abundance peaks of *O*. *similis* occurred in September (Fig. 8), despite different environmental conditions in both years of the study, driven mostly by different intensity of Atlantic waters inflow and slightly different dynamics of primary production blooms. Nevertheless elevated abundance of *O*. *similis* was associated with the highest temperature recorded in each year of study. A relatively high population of the Atlantic indicator–*O*. *atlantica* was also observed during this time. *Oithona* was a dominating copepod at the time when larger *Calanus* species descended for winter diapause. The high sex ratio observed twice a year during periods of high primary production strongly suggests that for this species there are two main reproductive events per year in the high Arctic fjord. *O*. *similis* seemed to actively reproduced even under low temperatures (< 0 °C), which is in contradiction with the previously indicated temperature < 5 °C as a factor significantly limiting its fecundity and distribution. Our research adds to the evidence that opportunistic species such as *O*. *similis* due to its unique thermal tolerance and ecological plasticity are likely to take advantage of the niche range extension resulting from an intensified period of Atlantification in the Arctic region.

## Supplementary Information

Supplementary Information.

## Data Availability

The datasets analysed during the current study are available from the corresponding author on request.

## References

[1] Descamps S (2017). Climate change impacts on wildlife in a High Arctic archipelago–Svalbard, Norway. Glob. Change Biol..

[2] Yletyinen J (2019). Arctic climate resilience. Nat. Clim. Change.

[3] Renaud PE (2018). Pelagic food-webs in a changing Arctic: A trait-based perspective suggests a mode of resilience. ICES J. Mar. Sci..

[4] Möller EF, Nielsen TG (2020). Borealization of Arctic zooplankton—smaller and less fat zooplankton species in Disko Bay, Western Greenland. Limnol. Oceanogr..

[5] Dalpadado P (2020). Climate effects on temporal and spatial dynamics of phytoplankton and zooplankton in the Barents Sea. Prog. Oceanogr..

[6] Csapó HK, Grabowski M, Węsławski JM (2021). Coming home - Boreal ecosystem claims Atlantic sector of the Arctic. Sci. Total. Environ..

[7] Bauerfeind E, Nöthig EM, Pauls B, Kraft A, Beszczynska-Möller A (2014). Variability in pteropod sedimentation and corresponding aragonite flux at the Arctic deep-sea long-term observatory HAUSGARTEN in the eastern Fram Strait from 2000 to 2009. J. Mar. Syst..

[8] Weydmann A (2014). Shift towards the dominance of boreal species in the Arctic: Inter-annual and spatial zooplankton variability in the West Spitsbergen Current. Mar. Ecol. Prog. Ser..

[9] Gluchowska M (2016). Zooplankton in Svalbard fjords on the Atlantic-Arctic boundary. Polar. Biol..

[10] Wassmann P (2015). The contiguous domains of Arctic Ocean advection: Trails of life and death. Prog. Oceanogr..

[11] Nielsen TG, Andersen C (2002). Plankton community structure and production along a freshwater-influenced Norwegian fjord system. Mar. Biol..

[12] Lischka S, Hagen W (2005). Life histories of the copepods Pseudocalanus minutus, *P*.* acuspes*, (Calanoida) and *Oithona similis* (Cyclopoida) in the Arctic Kongsfjorden (Svalbard). Polar Biol..

[13] Arendt KE, Nielsen TG, Rysgaard S, Tönnesson K (2010). Differences in plankton community structure along the Godthåbsfjord, from the Greenland Ice Sheet to offshore waters. Mar. Ecol. Prog. Ser..

[14] Trudnowska E, Stemmann L, Błachowiak-Samołyk K, Kwasniewski S (2020). Taxonomic and size structures of zooplankton communities in the fjords along the Atlantic water passage to the Arctic. J. Mar. Sys..

[15] Balazy K, Trudnowska E, Wichorowski M, Błachowiak-Samołyk K (2018). Large versus small zooplankton in relation to temperature in the Arctic shelf region. Polar. Res..

[16] Turner JT (2004). The importance of small planktonic copepods and their roles in pelagic marine food webs. Zool. Stud..

[17] Turner JT (1994). Planktonic copepods of Boston Harbor, Massachusetts Bay and Cape Cod Bay. Hydrobiologia.

[18] Castellani C, Robinson C, Smith T, Lampitt RS (2005). Temperature affects respiration rate of *Oithona similis*. Mar. Ecol. Prog. Ser..

[19] Turner JT, Levinsen H, Nielsen TG, Hansen BW (2001). Zooplankton feeding ecology: Grazing on phytoplankton and predation on protozoans by copepod and barnacle nauplii in Disko Bay, West Greenland. Mar. Ecol. Prog. Ser..

[20] Boissonnot L, Niehoff B, Hagen W, Søreide JE, Graeve M (2016). Lipid turnover reflects life-cycle strategies of small-sized Arctic copepods. J. Plankton Res..

[21] Błachowiak-Samołyk K (2015). Winter Tales: The dark side of planktonic life. Polar Biol..

[22] Berge J, Berge J, Johnsen G, Cohen J (2020). Zooplankton in the Polar Night in Polar Night Marine Ecology. Advances in Polar Ecology.

[23] Hobbs L, Banas NS, Cottier FR, Berge J, Daase M (2020). Eat or sleep: Availability of winter prey explains mid-winter and early-spring activity in an Arctic *Calanus* population. Front. Mar. Sci..

[24] Svensen C, Seuthe L, Vasilyeva Y, Pasternak A, Hansen E (2011). Zooplankton distribution across Fram Strait in autumn: Are small copepods and protozooplankton important?. Prog. Oceanog..

[25] Węsławski JM, Kwasniewski S, Wiktor J (1991). Winter in Svalbard fjord ecosystem. Arctic.

[26] Lischka S, Giménez L, Hagen W, Ueberschär B (2007). Seasonal changes in digestive enzyme (trypsin) activity of the copepods *Pseudocalanus minutus* (Calanoida) and *Oithona similis* (Cyclopoida) in the Arctic Kongsfjorden (Svalbard). Polar Biol..

[27] Lischka S, Hagen W (2016). Seasonal dynamics of mesozooplankton in the Arctic Kongsfjord (Svalbard) during year-round observations from August 1998 to July 1999. Polar Biol..

[28] Weydmann-Zwolicka A (2021). Zooplankton and sediment flux in two contrasting fjords reveal Atlantification of the Arctic. Sci. Total. Environ..

[29] Zamora-Terol S, Nielsen TG, Saiz E (2013). Plankton community structure and role of *Oithona similis* on the western coast of Greenland during the winter-spring transition. Mar. Ecol. Prog. Ser..

[30] Zamora-Terol S, Kjellerup S, Swalethorp R, Saiz E, Nielsen TG (2014). Population dynamics and production of the small copepod *Oithona* spp. in a subarctic fjord of West Greenland. Polar. Biol..

[31] Dvoretsky VG, Dvoretsky AG (2009). Life cycle of *Oithona similis* (Copepoda: Cyclopoida) in Kola Bay (Barents Sea). Mar. Biol..

[32] Glad, P. Seasonal occurrence of *Oithona similis* (cyclopoida), *Microsetella norvegica* (harpacticoida) and *Microcalanus* spp. (calanoida), and productivity of *O*. *similis*, in three high-latitude Norwegian fjords. Master thesis (UiT The Arctic University of Norway, 2018).

[33] Kosobokova K, Hirche HJ (2009). Biomass of zooplankton in the eastern Arctic Ocean—a baseline study. Progr. Oceanogr..

[34] Bluhm B, Kosobokova K, Carmack E (2015). A tale of two basins: An integrated physical and biological perspective of the deep Arctic Ocean. Prog. Oceanog..

[35] Hop H, Hop H, Wiencke C (2019). Zooplankton in Kongsfjorden (1996–2016) in Relation to Climate Change in *The Ecosystem of Kongsfjorden, Svalbard*. Advances in Polar Ecology.

[36] Böttger-Schnack R, Schnack D, Hagen W (2008). Microcopepod community structure in the Gulf of Aqaba and northern Red Sea, with special reference to Oncaeidae. J. Plankton Res..

[37] Cornwell LE (2018). Seasonality of *Oithona similis* and *Calanus helgolandicus* reproduction and abundance: Contrasting responses to environmental variation at a shelf site. J. Plankton Res..

[38] Kubiszyn AM (2017). The annual planktonic protist community structure in an ice-free high Arctic fjord (Adventfjorden, West Spitsbergen). J. Mar. Syst..

[39] Kellogg CTE, McClelland JW, Dunton KH, Crump BC (2019). Strong seasonality in Arctic estuarine microbial food webs. Front. Microbiol..

[40] Bhaskar JT, Parli BV, Tripathy SC (2020). Spatial and seasonal variations of dinoflagellates and ciliates in the Kongsfjorden. Svalbard. Mar. Ecol..

[41] Skogseth R (2020). Variability and decadal trends in the Isfjorden (Svalbard) ocean climate and circulation–An indicator for climate change in the European Arctic. Prog. Oceanog..

[42] Ward P, Hirst AG (2007). *Oithona similis* in a high latitude ecosystem: Abundance, distribution and temperature limitation of fecundity rates in a sac spawning copepod. Mar. Biol..

[43] Nielsen TG, Sabatini M (1996). Role of cyclopoid copepods Oithona spp. in North Sea plankton communities. Mar. Ecol. Prog. Ser..

[44] Nilsen F, Cottier F, Skogseth R, Mattsson S (2008). Fjord–shelf exchanges controlled by ice and brine production: The interannual variation of Atlantic Water in Isfjorden, Svalbard. Cont. Shelf Res..

[45] Cohen JH, Berge J, Moline MA, Johnsen G, Zolich AP, Berge J, Johnsen G, Cohen J (2020). Light in the Polar Night. Polar Night Marine Ecology Advances in Polar Ecology.

[46] Wiedmann I, Reigstad M, Marquardt M, Vader A, Gabrielsen TM (2015). Seasonality of vertical flux and sinking particle characteristics in an ice-free high arctic fjord—different from subarctic fjords?. J. Mar. Syst..

[47] Holm-Hansen O, Riemann B (1978). Chlorophyll a determination: Improvements in methodology. Oikos.

[48] Stübner EI, Søreide JE, Reigstad M, Marquardt M, Blachowiak-Samolyk K (2016). Year-round meroplankton dynamics in high-Arctic Svalbard. J. Plankton Res..

[49] Marquardt M, Vader A, Stübner EI, Reigstad M, Gabrielsen TM (2016). Strong seasonality of marine microbial eukaryotes in a high-Arctic fjord (Isfjorden, West Spitsbergen). Appl. Environ. Microb..

[50] Trantner, D. J. & Fraser, H. Zooplankton sampling. Monographs on Oceanographic Methodology 2. (UNESCO, 1968).

[51] Harris R, Wiebe L, Lenz J, Skjoldal HR, Huntley M (2000). ICES Zooplankton Methodology Manual.

[52] Espinasse M (2018). Interannual phenological variability in two North-East Atlantic populations of *Calanus finmarchicus*. Mar. Biol. Res..

[53] Mackas DL, Batten S, Trudel M (2007). Effects on zooplankton of a warmer ocean: Recent evidence from the Northeast Pacific. Prog. Oceanogr..

[54] Head EJH, Melle W, Pepin P, Bagøien E, Broms C (2013). On the ecology of *Calanus finmarchicus* in the Subarctic North Atlantic: A comparison of population dynamics and environmental conditions in areas of the Labrador Sea-Labrador/Newfoundland Shelf and Norwegian Sea Atlantic and Coastal Waters. Prog. Oceanog..

[55] Kwasniewski S (2012). Interannual changes in zooplankton on theWest Spitsbergen Shelf in relation to hydrography and their consequences for the diet of planktivorous seabirds. J. Mar. Sci..

[56] Kiorboe T (2006). Sex, sex-ratios, and the dynamics of pelagic copepod populations. Oecol..

[57] Thackeray (2013). Food web de-synchronization in England’s largest lake: An assessment based on multiple phenological metrics. Glob. Change Biol..

[58] Anderson, M. J., Gorley, R. N. & Clarke, K. R. *PERMANOVA+ for PRIMER: Guide to Software and Statistical Methods*. (Primer-E Ltd., 2008).

[59] Clarke, K. R. & Gorley, R. N. Primer. (Primer-E Ltd., 2001).

[60] Anderson MJ, Braak CJF (2003). Permutation tests for multi-factorial analysis of variance. J. Stat. Comput. Simul..

[61] Schlitzer, R. Ocean Data View; https://odv.awi.de, (2021).

[62] Walczowski W, Piechura J, Goszczko I, Wieczorek P (2012). Changes in Atlantic water properties: An important factor in the European Arctic marine climate. ICES J. Mar. Sci.

[63] Wassman P, Duarte CM, Agustí S, Sejr ML (2010). Footprints of climate change in the Arctic marine ecosystem. Glob. Change Biol..

[64] Andrews AJ (2019). Boreal marine fauna from the Barents Sea disperse to Arctic Northeast Greenland. Sci Rep.

[65] Atkinson D, Sibly RM (1997). Why are organisms usually bigger in colder environments? Making sense of life history puzzle. Trends Ecol. Evol..

[66] Beaugrand G, Ibanez F, Reid PC (2000). Spatial seasonal and long term fluctuations of plankton in relation to hydroclimatic features in the English Channel, Celtic Sea and Bay of Biscay. Mar. Ecol. Prog. Ser..

[67] Beaugrand G, Reid PC, Ibañez F, Lindley A, Edwards M (2002). Reorganization of North Atlantic Marine Copepod Biodiversity and Climate. Science.

[68] Coyle KO (2011). Climate change in the southeastern Bering Sea: Impacts on pollock stocks and implications for the oscillating control hypothesis. Fisher. Oceanogr..

[69] Edwards M, Richardson AJ (2004). The impact of climate change on the phenology of the plankton community and trophic mismatch. Nature.

[70] Stevens GC (1989). The latitudinal gradient in geographical range: How so many species coexist in the tropics. Am. Nat..

[71] Kortsch S, Primicerio R, Fossheim M, Dolgov AV, Aschan M (2015). Climate change alters the structure of arctic marine food webs due to poleward shifts of boreal generalists. Proc. R. Soc. B..

[72] Richardson AJ (2008). In hot water: Zooplankton and climate change. ICES J. Mar. Sci..

[73] Kwasniewski S (1990). A note on zooplankton of the Hornsund Fjord and its seasonal changes. Oceanografia.

[74] Piwosz K (2009). Comparison of productivity and phytoplankton in a warm (Kongsfjorden) and a cold (Hornsund) Spitsbergen fjord in midsummer 2002. Polar Biol..

[75] Trudnowska E, Basedow SL, Blachowiak-Samolyk K (2014). Mid-summer mesozooplankton biomass, its size distribution, and estimated production within a glacial Arctic fjord (Hornsund, Svalbard). J. Mar. Syst..

[76] Castellani C, Licandro P, Fileman E, di Capua I, Mazzocchi MG (2016). *Oithona similis* likes it cool: Evidence from two long-term time series. J. Plankton Res..

[77] Cornwell LE (2020). Resilience of the copepod *Oithona similis* to climatic variability: Egg production, mortality, and vertical habitat partitioning. Front. Mar. Sci..

[78] Eiane K, Ohman MD (2004). Stage-specific mortality of *Calanus finmarchicus*, *Pseudocalanus elongatus* and *Oithona similis* on Fladen Ground, North Sea, during a spring bloom. Mar. Ecol. Prog. Ser..

[79] Thor P (2005). Post-spring bloom community structure of pelagic copepods in the Disko Bay, Western Greenland. J. Plankton Res..

[80] Dvoretsky VG (2012). Seasonal mortality rates of *Oithona similis* (Cyclopoida) in a large Arctic fjord. Polar Sci..

[81] Ussing, H. H. The biology of some important plankton animals in the fjords of east Greenland. *Medd Grønland* 100–108 (1938).

[82] Lonsdale DJ, Caron DA, Dennett MR, Schaffner R (2000). Predation by Oithona spp on protozooplankton in the Ross Sea. Antarctica. Deep-Sea Res. II.

[83] Castellani C, Irigoien X, Harris RP, Lampitt RS (2005). Feeding and egg production of *Oithona similis* in the North Atlantic. Mar. Ecol. Prog. Ser..

[84] Barth-Jensen C (2020). Temperature-dependent egg production and egg hatching rates of small egg-carrying and broadcast-spawning copepods *Oithona similis*, *Microsetella norvegica* and *Microcalanus pusillus*. J. Plankton Res..

[85] Falk-Petersen S, Pedersen G, Kwasniewski S, Hegseth EN, Hop H (1999). Spatial distribution and life-cycle timing of zooplankton in the marginal ice zone of the Barents Sea during the summer melt season in 1995. J. Plankton Res..

[86] Gluchowska M (2017). Interannual zooplankton variability in the main pathways of the Atlantic water flow into the Arctic Ocean (Fram Strait and Barents Sea branches). ICES J. Mar. Sci..

[87] Balazy K, Trudnowska E, Błachowiak-Samołyk K (2019). Dynamics of *Calanus* copepodite structure during Little Auks’ breeding seasons in two different Svalbard locations. Water.

[88] Hop H (2002). The marine ecosystem of Kongsfjorden, Svalbard. Polar Res..

[89] Poje, A. The relationship between plankton and water mass properties in high Arctic (Svalbard) fjords. Clark Honors College Theses, (University of Oregon, 2016).

[90] Falk-Petersen S, Mayzaud P, Kattner G, Sargent JR (2009). Lipids and life strategy of Arctic *Calanus*. Mar. Biol. Res..

[91] Svensen C (2019). Zooplankton communities associated with new and regenerated primary production in the Atlantic inflow North of Svalbard. Front. Mar. Sci..

[92] González HE, Smetacek V (1994). The possible role of the cyclopoid copepod Oithona in retarding vertical flux of zooplankton faecal material. Mar. Ecol. Prog. Ser..

[93] Berge J (2015). Unexpected levels of biological activity during the polar night offer new perspectives on a warming Arctic. Curr. Biol..

[94] Berge J (2015). In the dark: A review of ecosystem processes during the Arctic polar night. Progr. Oceanog..

[95] Narcy F (2009). Seasonal and individual variability of lipid reserves in *Oithona similis* (Cyclopoida) in an Arctic fjord. Polar Biol..

[96] Kattner G, Hagen W, Kainz M, Brett MT, Arts MT (2009). Lipids in marine copepods: Latitudinal characteristics and perspective to global warming. Lipids in Aquatic Ecosystems.

[97] Rokkan Iversen K, Seuthe L (2011). Seasonal microbial processes in a high-latitude fjord (Kongsfjorden, Svalbard): I. Heterotrophic bacteria, picoplankton and nanoflagellates. Polar Biol..

[98] Auel H, Hagen W (2002). Mesozooplankton community structure, abundance and biomass in the central Arctic Ocean. Mar. Biol..

[99] Madsen S, Nielsen T, Hansen B (2008). Annual population development and production by small copepods in Disko Bay, western Greenland. Mar. Biol..

[100] Corkett CJ, McLaren IA, Russell FS, Yonge M (1978). The biology of *Pseudocalanus*. Advances in Marine Biology.

[101] Kwasniewski S, Hop H, Falk-Petersen S, Pedersen G (2003). Distribution of *Calanus* species in Kongsfjorden, a glacial fjord in Svalbard. J. Plankton Res..

[102] Willis K, Cottier F, Kwasniewski S, Wold A, Falk-Petersen S (2006). The influence of advection on zooplankton community composition in an Arctic fjord (Kongsfjorden, Svalbard). J. Mar. Syst..

