# Supplementary material for: Phenology of Oithona similis demonstrates that ecological flexibility may be a winning trait in the warming Arctic
Source: Sci Rep. 2021 Sep 20;11:18599. doi: 10.1038/s41598-021-98068-8 (PMC8452673; doi:10.1038/s41598-021-98068-8)
Supplement: Supplementary file 1 — Supplementary Information. [file 41598_2021_98068_MOESM1_ESM.pdf]

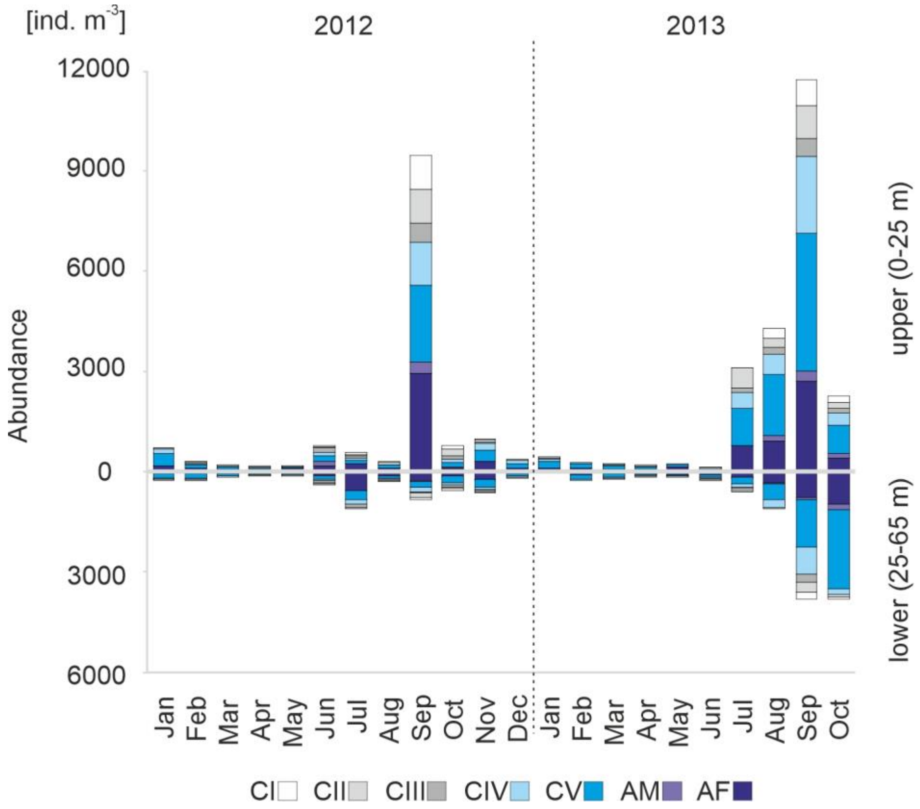

Fig. S1 *Oithona similis* copepodite stages (CI-CV, AF – adult females, AM – adult males) monthly mean abundance measured at IsA station in two water layers: upper (0-25 m) and lower (25-65 m).

Table S1. Results of the DistLM analysis for fitting environmental variables to *O. similis* total abundance, sex ratio (AM/AF), average weighted stage (AWS), proportion of CV to total abundance (CVT), population development index (PDI). Var% - percentage of explained variance; Cum% - cumulative percentage explained by the added variable. Variables with statistically significant impact, with p values <0.005 were bolded.

	Marginal tests				Sequential tests					
	Variable	Pseudo-F	*P*	Var%	Variable	R^2	Pseudo-F	*P*	Var%	Cum%
**Total ab.**	Salinity	3.1	0.073	0.13	**+Temperature**	0.62	32.40	**0.001**	0.62	0.62
	**Temperature**	32.4	**0.001**	0.62	+Daylength	0.65	1.55	0.225	0.03	0.65
	Chl *a*	1.21	0.288	0.06	+*Calanus* spp.	0.70	2.76	0.074	0.05	0.69
	Daylength	0.26	0.749	0.01	+Salinity	0.74	2.75	0.071	0.04	0.74
	Calanus spp.	0.74	0.435	0.04	+Chl *a*	0.74	0.56	0.580	<0.01	0.74
**AM/AF**	Salinity	0.06	0.882	<0.01	**+Chl *a***	0.43	14.77	**0.002**	0.42	0.42
	Temperature	1.83	0.172	0.08	**+Temperature**	0.56	6.11	**0.018**	0.14	0.56
	**Chl *a***	14.77	**0.002**	0.43	+Daylength	0.60	1.75	0.209	0.04	0.60
	**Daylength**	3.76	**0.050**	0.15	+Salinity	0.61	0.48	0.498	0.01	0.61
	Calanus spp.	0.10	0.780	<0.01	+*Calanus* spp.	0,62	0.20	0.698	<0.01	0.62
**AWS**	**Salinity**	5.27	**0.035**	0.21	**+Temperature**	0.27	7.31	**0.008**	0.27	0.27
	**Temperature**	7.31	**0.011**	0.27	+Salinity	0.36	2.66	0.107	0.09	0.36
	Chl *a*	1.62	0.228	0.07	+Chl *a*	0.41	1.72	0.199	0.06	0.41
	Daylength	0.08	0.782	<0.01	+Daylength	0.42	0.23	0.628	<0.01	0.42
	Calanus spp.	0.07	0.804	<0.01	+*Calanus* spp.	0.42	0.01	0.919	<0.01	0.42
**CVT**	Salinity	0.74	0.418	0.04	**+Chl *a***	0.43	15.19	**0.004**	0.43	0.43
	Temperature	0.08	0.809	<0.01	+*Calanus* spp.	0.47	1.56	0.202	0.04	0.47
	**Chl *a***	15.19	**0.002**	0.43	+Salinity	0.50	0.82	0.399	0.02	0.50
	**Daylength**	10.624	**0.001**	0.35	+Temperature	0.50	0.19	0.689	<0.01	0.50
	Calanus spp.	2.24	0.108	0.10	+Daylength	0.50	0.08	0.792	<0.01	0.50
**PDI**	**Salinity**	5.74	**0.020**	0.22	**+Temperature**	0.31	9.07	**0.005**	0.31	0.31
	**Temperature**	9.07	**0.008**	0.31	+Salinity	0.40	2.90	0.093	0.09	0.40
	Chl *a*	<0.01	0.981	<0.01	+*Calanus* spp.	0.47	2.20	0.172	0.06	0.47
	Daylength	2.42	0.124	0.11	+Daylength	0.47	0.13	0.720	<0.01	0.47
	Calanus spp.	2.14	0.169	0.10	+Chl *a*	0.48	0.27	0.620	<0.01	0.48
